# Metastatic lymph node targeted CTLA4 blockade: a potent intervention for local and distant metastases with minimal ICI-induced pneumonia

**DOI:** 10.1186/s13046-023-02645-w

**Published:** 2023-06-01

**Authors:** Radhika Mishra, Ariunbuyan Sukhbaatar, Shiro Mori, Tetsuya Kodama

**Affiliations:** 1 Laboratory of Biomedical Engineering for Cancer, Graduate School of Biomedical Engineering, Tohoku University, 4-1 Seiryo, Aoba, Sendai, Miyagi 980-8575 Japan; 2 Biomedical Engineering Cancer Research Center, Graduate School of Biomedical Engineering, Tohoku University, 4-1 Seiryo, Aoba, Sendai, Miyagi 980-8575 Japan; 3 Division of Oral and Maxillofacial Oncology and Surgical Sciences, Graduate School of Dentistry, Tohoku University, 4-1 Seiryo, Aoba, Sendai, Miyagi 980-8575 Japan

**Keywords:** CTLA4, Local immune checkpoint blockade, Metastatic lymph node, Immune related adverse events, Interstitial pneumonia

## Abstract

**Background:**

Immune checkpoint blockade (ICB) elicits a strong and durable therapeutic response, but its application is limited by disparate responses and its associated immune-related adverse events (irAEs). Previously, in a murine model of lymph node (LN) metastasis, we showed that intranodal administration of chemotherapeutic agents using a lymphatic drug delivery system (LDDS) elicits stronger therapeutic responses in comparison to systemic drug delivery approaches, while minimizing systemic toxicity, due to its improved pharmacokinetic profile at the intended site. Importantly, the LN is a reservoir of immunotherapeutic targets. We therefore hypothesized that metastatic LN-targeted ICB can amplify anti-tumor response and uncouple it from ICB-induced irAEs.

**Methods:**

To test our hypothesis, models of LN and distant metastases were established with luciferase expressing LM8 cells in MXH10/Mo-*lpr/lpr* mice, a recombinant inbred strain of mice capable of recapitulating ICB-induced interstitial pneumonia. This model was used to interrogate ICB-associated therapeutic response and immune related adverse events (irAEs) by in vivo imaging, high-frequency ultrasound imaging and histopathology. qPCR and flowcytometry were utilized to uncover the mediators of anti-tumor immunity.

**Results:**

Tumor-bearing LN (tbLN)-directed CTLA4 blockade generated robust anti-tumor response against local and systemic metastases, thereby improving survival. The anti-tumor effects were accompanied by an upregulation of effector CD8T cells in the tumor-microenvironment and periphery. In comparison, non-specific CTLA4 blockade was found to elicit weaker anti-tumor effect and exacerbated ICI-induced irAEs, especially interstitial pneumonia. Together these data highlight the importance of tbLN-targeted checkpoint blockade for efficacious response.

**Conclusions:**

Intranodal delivery of immune checkpoint inhibitors to metastatic LN can potentiate therapeutic response while minimizing irAEs stemming from systemic lowering of immune activation threshold.

**Supplementary Information:**

The online version contains supplementary material available at 10.1186/s13046-023-02645-w.

## Background

Lymph node (LN) metastasis is a common occurrence in various malignancies and is often indicative of an endpoint event [1–5]. Current standard care includes lymphadenectomy or systemic chemotherapy [3]. However, lymphadenectomy can lead to activation of latent tumor cells at distant sites, making it counterproductive [6–8]. Additionally, efficient drug delivery to metastatic LNs (MLNs) by systemic drug delivery approaches is challenging due to the existence of a unique set of drug transport barriers [9, 10].

Immune checkpoint blockade (ICB) has revolutionized the world of cancer therapeutics. Abrogation of negative immune feedback provided by the upregulation of CTLA4 on T cells and PD1 on B cells, T cells, and several other cells results in a durable anti-tumor response [11, 12]. Presently, seven PD1/PDL1 inhibitors, and two CTLA4 inhibitors are FDA approved for monotherapy or combination use in various settings. Astoundingly, a fraction of patients is unresponsive to ICB [13, 14]. Cases of hyperprogression have also been observed [15–17]. Particularly, with CTLA4 blockers, progress has been slow due to its complex rheostat like biology [18], with FDA approval being limited to melanoma, and in combination with nivolumab, for renal cell carcinoma, metastatic colorectal cancer, hepatocellular carcinoma, non-small cell lung cancer and pleural mesothelioma [11, 19]. However, at present greater than 100 clinical trials and multiple preclinical studies are underway to expand its clinical use [20].

Previously we have shown that intranodal administration of chemotherapeutic agents using an ultrasound-guided lymphatic drug delivery system (LDDS) significantly improves drug pharmacokinetics and is thus a promising strategy for the treatment of MLNs [9, 21]. Additionally, LN is a reservoir of immunotherapeutic targets. Importantly, it is the site of antigen-presentation and immune activation [22]. LN-targeting could provide an opportunity to modify adaptive immune responses. Therefore, we hypothesized that immune checkpoint blockade, specifically in the tumor-bearing LN (tbLN), unlike systemic immune checkpoint blockade, can potentiate anti-tumor response. Particularly, we were interested in anti-CTLA4 as, CTLA4 checkpoint is known to primarily act in the initial stages of naïve T cell activation, typically in the lymph nodes unlike anti-PD1, that primarily acts upon previously activated T cells at later stages of T cell response, primarily in the peripheral tissue [11, 23–25].

Intranodal administration of immune-modulating agents is a dimension that has previously been explored [22, 26–30]. However, clinical data is mostly limited to dendritic cell (DC) based vaccines or mRNA vaccines relying on the antigen-presenting role of DCs [28, 30]. Bedrosian et. al. showed superior T cell sensitization upon intranodal administration of peptide-pulsed mature dendritic cell vaccines [26]. Morisaki et. al. showed clinical and immunological promise of intranodaly administered neoantigen peptide loaded DCs [27]. Common to these studies was a significant upregulation of functional T cells mediated by highly populous DCs in LN. Kreiter et. al. showed for the first time, in a pre-clinical model, a shift to Th1-type immunity with naked antigen-encoding RNA [28]. Importantly, these studies demonstrated the untapped potential of LN targeted immunomodulation.

However, to the best of our knowledge, there are no studies assessing the consequences of intranodal administration of immune checkpoint blockade [31], particularly in the unique immune landscape of MLN. Therefore, we sought to verify our hypothesis in a mouse model of lymph node metastasis.

Apart from dismal responses in a swath of patients, another factor limiting the widespread utility of ICIs is the manifestation of immune related adverse events (irAEs) caused by the lowering of the threshold for T cell activation [32–34]. Development of severe irAEs often leads to a stop or delay in treatment, facilitating cancer progression [13, 35, 36]. However, currently, robust models recapitulating clinically observed ICI monotherapy induced irAEs are lacking [35, 37]. Particularly, ICI-induced interstitial lung disease is an event of special interest as it is associated with high morbidity and mortality [38–42]. However, due to the absence of relevant pre-clinical models, a clear understanding of its pathogenesis is lacking. Additionally, literature describing ICI-induced interstitial lung disease is limited. Therefore, it is imperative to develop suitable in vivo models capable of recapitulating clinically observed ICI-induced pneumonia to facilitate studies uncovering the mechanistic underpinnings of ICB associated irAEs. This will be critical to designing effective strategies to minimize, if not uncouple, therapeutic response from irAE, particularly in the context of combination therapies where dose-limiting toxicity has restricted its widespread utility [22].

So, to successfully replicate clinically observed ICI monotherapy-induced irAEs, we utilized a recombinant inbred strain of mice, MXH10/Mo-*lpr/lpr* (MXH10/Mo/lpr), derived from MRL/Mp-*lpr/lpr* (MRL/lpr) and C3H/HeJ-*lpr/lpr*. These mice exhibit systemic lymphadenopathy, due to the Fas deletion mutant, bearing LNs of approximately 10 mm in diameter at 10 – 12 weeks of age, facilitating reliable experimental manipulations of the murine lymphatic network. Importantly, unlike its ancestor, the MRL/lpr mouse, it does not spontaneously develop fatal collagen diseases [43–46]. It, however, shares some of the background genes of MRL/lpr mouse (Table S1), making it susceptible to autoimmune conditions upon specific insults. Thus, MXH10/Mo/lpr successfully recapitulates clinical manifestations of certain ICI-induced irAEs, as shown in the present study. Recent reports support the possibility of genetic susceptibility to irAE as one of the risk factors to ICI-induced irAEs [47–49]. Thus, MXH10/Mo/lpr is a suitable model to study to the consequences of immune checkpoint blockade and is critical to the understanding of ICI-induced irAEs, which in turn can inform the design of personalized therapeutics to ameliorate ICI-induced irAEs or uncouple them from the therapeutic response.

Herein, we present, for the first time, a simple model for ICI-induced interstitial pneumonia, investigations in which provide critical insights that may help overcome critical bottlenecks to the advancement of cancer immunotherapy.

## Methods

### Mice

MXH10/Mo/lpr mice were bred under pathogen-free conditions at Animal Research Institute, Tohoku University. Experiments utilized LNs in the subiliac and axillary regions, the subiliac LN (SiLN) and the proper axillary LN (PALN), respectively, to study lymph node metastasis. All experimentations were initiated at 12 weeks of age and were in accordance with the guidelines of the Institutional Animal Care and Use Committee of Tohoku University.

### Cell line

LM8-Luc cells, an aggressively metastatic cell line derived from Dunn’s osteosarcoma were cultured in DMEM (Dulbecco’s Modified Eagle Medium, Sigma Aldrich, St. Louis, MO, USA) supplemented with 10% FBS-supplemented (Fetal Bovine Serum, HyClone Laboratories Inc., UT, USA), 1% L-glutamine-penicillin–streptomycin (Sigma Aldrich, St. Louis, MO, USA) and 1 mg/mL Geneticin (G418 Fujifilm Wako Pure Chemical Industries, Osaka, Japan) and maintained at 37 °C and 5% CO_2_. Cells were checked for mycoplasma contamination and passaged twice prior to inoculation.

### Tumor bearing LN mouse model

LM8-Luc cells (3.3 × 10^5^ cells/mL, 60 µL) in a 1:2 mixture of PBS (Sigma Aldrich, St. Louis, MO, USA) and Matrigel (Corning; Bedford, MA, USA), were unilaterally or bilaterally inoculated to the center of the SiLN of MXH10/Mo/lpr mice to establish murine models to facilitate study of metastatic LNs. Skin was depilated and an incision was made prior to inoculation to expose the SiLN.

### Immune checkpoint blockade

Anti-CTLA4 monoclonal antibody (9H10, Biolegend, San Diego, CA, USA), 5 mg/kg (cumulative dosage: 10 mg/kg), was administered to tumor-bearing SiLN (tbLN), or to non-tumor bearing SiLN (ntbLN) through a LDDS [9], or intraperitoneally, on day 4 and 8, to mice inoculated unilaterally to study the impact of drug administration strategy on anti-tumor response of ICI. For immunome characterization, mice treated as above were euthanized on day 9.

Having determined the optimal drug delivery methodology, 5 mg/kg anti-CTLA4 mAb was administered on day 4 and 8 to the right SiLN of mice harboring tumor in both the SiLNs, to evaluate whether the cancer immunotherapeutic effect was potent enough to limit distant metastasis by eliciting a systemic response.

Subsequently, to determine the sensitivity of therapeutic response to anti-CTLA4 dosage, a dose de-escalation study was performed. On day 4, 1 mg/kg or 5 mg/kg anti-CTLA4 mAb was administered to the tbLN through LDDS of unilaterally inoculated mice.

Then, therapeutic efficacy of locally delivered low dose intranodal anti-PD1 was investigated. Anti-PD1 mAb (CD279, 114,102, Biolegend, San Diego, CA, USA) 0.5 mg/kg or 1 mg/kg or 5 mg/kg or 10 mg/kg, was administered into the tbLN on day 4 of unilaterally inoculated mice.

ICI were diluted in saline to adjust concentration, if necessary. 200 μL of concentration adjusted drug was administered on specified days.

### Tumor progression

In vivo bioluminescent imaging was performed using IVIS (IVIS; PerkinElmer Waltham, MA, USA) for quantification of tumor growth, biweekly for the first 3 weeks, and once a week for the next 3 weeks (day 4, 8, 11, 15, 18, 21, 28, 35 and 42). In addition, SiLN volumes were measured using high-frequency ultrasound device, VEVO770 (FUJIFILM VisualSonics, Tokyo, Japan) with a 40 MHz transducer (704B; VisualSonics). ex vivo bioluminescent imaging of SiLN, PALN, lung and liver was performed at the pre-determined experimental endpoint to investigate systemic metastasis.

In vivo luciferase activity and LN volumes were normalized to their day 4 and day 0 values, respectively for graphical representation. Complete response rates of individuals were confirmed by examination of stained tissue sections or bioluminescent imaging; Scoring – Complete response:1, Tumor still present at predetermined experimental endpoint:0. Incidence of liver and lung metastases were scored as follows for graphical representation; Metastasis detected:1; No metastasis detected:0.

### Terminal histopathology

Tissues harvested on day 42, or 21, for groups with low survival, or upon death, were molded into paraffin blocks. 4 μm thick sections of each were stained by hematoxylin and eosin (HE) or elastica Masson (EM) staining to examine tumor proliferation and the incidence of irAEs. Histological evaluation was done in a blinded fashion and grading for glomerulonephritis, vasculitis, sialadenitis was performed as described previously [44, 45, 50]. Evaluation for interstitial pneumonia was performed as follows: grade 0 (score:0), no pneumonia; grade 1 (score:1), focal interstitial pneumonia; grade 2 (score:2), multifocal pneumonia; grade 3a (score:3), diffuse interstitial pneumonia with partial loss of alveolar space in lobe; grade 3b (score:4), diffuse interstitial pneumonia with extensive loss of alveolar space in lobe (Fig. S1, Table S2). Sum of grades divided by the total number of sections evaluated was defined as the severity index.

### Spleen index

Spleen index is a measure indicative of spleen atrophy and is reflective of the immune function [51]. At predetermined experimental endpoint, mouse body weight was recorded. Post sampling, spleen weight was recorded for evaluation of spleen index. It was calculated according to the following formula:

$$\mathrm{spleen}\hspace{1mm}\mathrm{index}\left(\mathrm{unitless}\right)=\mathrm{spleen}\hspace{1mm}\mathrm{weight}\left(\mathrm g\right)\;\div\;\mathrm{mouse}\hspace{1mm}\mathrm{body}\hspace{1mm}\mathrm{ weight}\left(\mathrm g\right)$$

### Lymph node and peripheral immunome characterization

Single cell suspensions of SiLN and spleen samples of untreated and anti-CTLA4 treated mice (cumulative dose: 10 mg/kg through LDDS to tbLN or ntbLN, or i.p.) harvested on day 9 were prepared by mechanical digestion (gentleMACS™ Octo Dissociator, Miltenyi Biotec, Bergisch Gladbach, North Rhine-Westphalia, Germany). Erythrocyte (RBC) lysis was performed for spleen samples using RBC lysis solution 10x (Miltenyi Biotec, Bergisch Gladbach, North Rhine-Westphalia, Germany). Staining was performed using an antibody cocktail comprising of CD3 (17A2), CD8 (QA17A07), CTLA4 (UC10-4B9), PD1 (29F.1A12) and KLRG1 (2F1/KLRG1) from Biolegend, San Diego, CA, USA and CD4 (RM4.5) from BD Pharmingen, San Diego, CA, USA). Dead cells were excluded by inclusion of Zombie Aqua fixable viability kit (Biolegend, San Diego, CA, USA). All flowcytometric analyses were performed on Canto II Flowcytometer (BD Biosciences, Franklin Lakes, NJ, USA).

### Analysis of local and peripheral cytokine milieu

SiLN and spleen samples harvested on day 9 post tumor inoculation were homogenized using Vibra-Cell™ (Newtown, CT, USA) following which total RNA was extracted using FastGene® RNA Basic kit (Nippon Genetics, Tokyo, Japan). RNA was reverse transcribed using Applied Biosystems™ High-Capacity cDNA Reverse Transcription Kit (Waltham, MA, USA) following manufacturer’s instructions. Quantitative PCR of obtained samples was performed using Applied Biosystems 7500 Real-Time PCR System (Waltham, Massachusetts, United States) to quantify housekeeping gene *GAPDH, IL6, IL10, and INFγ* (all, Integrated DNA Technologies, Coralville, IA, USA) (table S3). mRNA quantification was performed using 2^−△△Ct^ method and log fold changes were computed for graphical representation.

### Statistical analysis

Statistical analyses were performed using Graphpad Prism 8.4.3 (GraphPad Software, La Jolla, CA, USA). Data is presented as mean ± standard error of the mean (S.E.M.) unless indicated otherwise. Data were subjected to ANOVA and Tukey’s post hoc test analyses to determine significant events. Statistical analyses for survival curves were performed by log-rank (Mantel-Cox) test. Two-sided *P* < 0.05 was considered a statistically significant finding and stars indicate the degree of significance (*P < 0.05. **P < 0.01, ***P < 0.001).

## Results

### ICI administration site is a critical variable dictating immune checkpoint therapy associated response and irAE

**Fig. 1:**
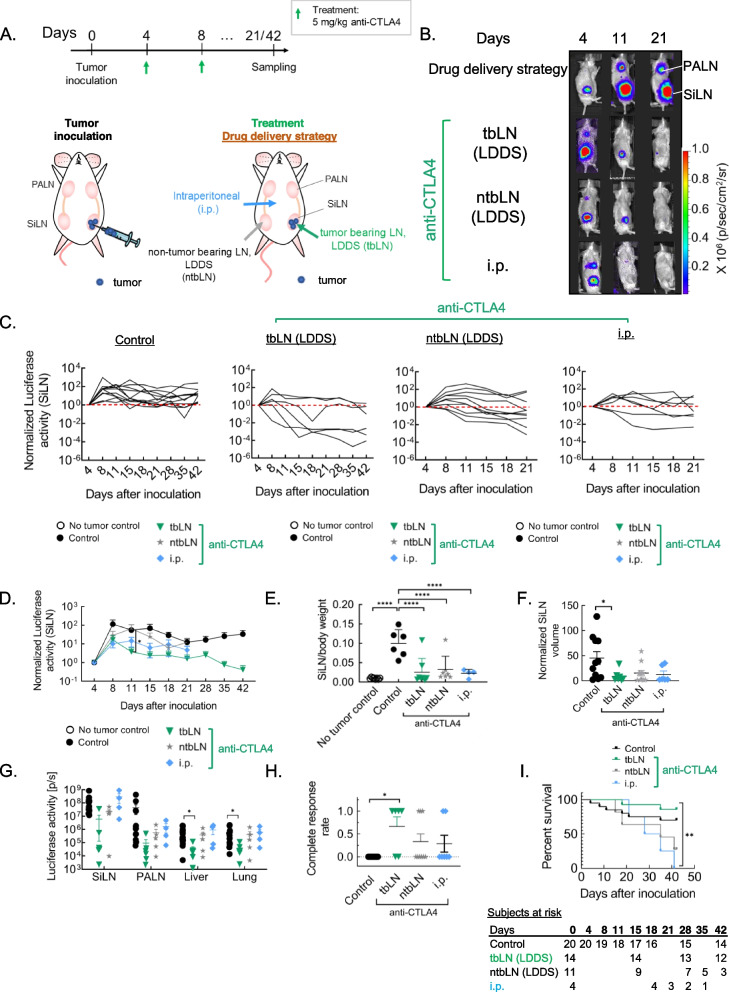
Delivery of anti-CTLA4 to tbLN using LDDS elicits robust and consistent tumor inhibitory response. 5 mg/kg a-CTLA4 mAb was administered into the tbLN or the ntbLN using a LDDS or through i.p. on day 4 and 8 post tumor inoculation to unilaterally inoculated MXH10/Mo/lpr mice to determine the optimal drug administration strategy. **A**. Experiment outline **B**. Representative in vivo luciferase activity **C**. Graphical representation of in vivo luciferase activity of the SiLN of individual mice **D**. Summarized graphical representation of normalized SiLN luciferase activity as a function of time. (**C** – **D**: Control, *n* = 12; tbLN, *n* = 6; ntbLN, *n* = 11; i.p., *n* = 7) **E**. SiLN weight normalized to mouse body weight (No tumor control, *n* = 8; Control, *n* = 6; tbLN, *n* = 8; ntbLN, *n* = 7; i.p., *n *= 4) **F**. Normalized SiLN volume as on the pre-determined experimental endpoint (Control, *n* = 11; tbLN, *n* = 7; ntbLN, *n* = 11; i.p., *n* = 6) **G**. ex vivo luciferase activity of the SiLN, PALN, liver and lung as measured on the pre-determined experimental endpoint (Control, *n* = 14; tbLN, *n* = 6; ntbLN, n = 5; i.p., *n* = 4) **H**. Complete response rates (Control, *n* = 6; tbLN, *n* = 13; ntbLN, *n* = 9; i.p., *n* = 7) **I**. Survival plots of mice (Control, *n* = 20; tbLN, *n* = 14; ntbLN, *n* = 11; i.p., *n* = 4). Statistical analyses were done using ANOVA with Tukey’s test. Log-rank (Mantel-Cox) test for survival curves. *P < 0.05, **P < 0.01, and ****P < 0.0001. Data are represented as means ± SEM

To determine the optimal ICI administration site, anti-CTLA4 mAb was administered as outlined (Fig. 1A). Therapeutic response was observed upon administration of anti-CTLA4 mAb through all drug delivery strategies. However, as opposed to other drug delivery methodologies, stronger, and consistent therapeutic response was observed upon localized delivery of anti-CTLA4 mAb to tbLN using a LDDS (Fig. 1B – H, Fig. S2). Notably, although incidence and severity of metastasis to lung in the control group itself was low (table S4), ex vivo luciferase activity of the liver and lung at endpoint were found to be significantly lower in these set of mice, indicating the potential of optimally dosed anti-CTLA4 mAb delivered to tbLN using LDDS to treat distant metastasis (Fig. 1G). Additionally, complete response rate was found to be markedly higher in these mice (tbLN: 4/6, ntbLN: 3/9, i.p.: 2/7) indicating that CTLA4 blockade, specifically in the tumor microenvironment (TME), was crucial for sustained tumor inhibition (Fig. 1H). Histological evaluation confirmed these findings (Fig. S2). Additionally, tbLN-directed CTLA4 blockade was also found to prolong overall survival. In stark contrast, overall survival for mice administered 10 mg/kg α-CTLA4 mAb via LDDS to ntbLN or i.p. was found to be staggeringly low despite apparent therapeutic benefit (Fig. 1 I).

**Fig. 2:**
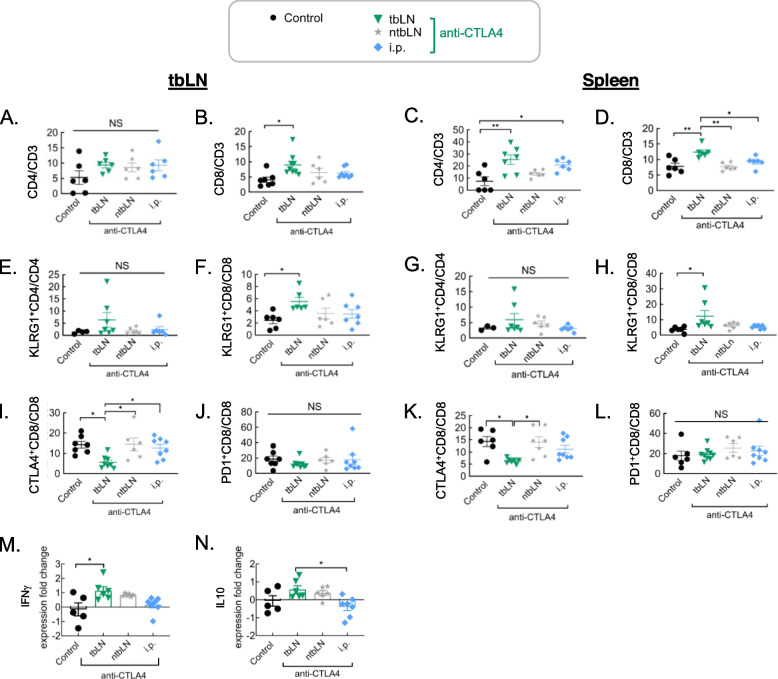
Intra-nodal administration to tbLN promotes tumor inhibiting immune microenvironment in the TME and periphery. Mice administered 5 mg/kg anti-CTLA4 mAb to the tbLN or the ntbLN using a LDDS or through i.p. on day 4 and 8 post unilateral tumor inoculation were euthanized on day 9. Subsequently the immune microenvironment of the tbLN or spleen was investigated. Frequencies of A. CD4T (all groups, *n* = 6) and B. CD8 (Control, *n* = 7; tbLN, *n* = 9; ntbLN, *n* = 6; i.p., *n *= 8) in the tbLN; Frequencies of C. CD4T (tbLN, *n* = 7; ntbLN, all other groups *n* = 6) and D. CD8 (all groups, *n* = 6) in the spleen; Frequencies of E. KLRG1^+^CD4T cells in the tbLN (Control, *n* = 4; tbLN,* n* = 7; ntbLN, *n* = 6; i.p., *n* = 6) and F. KLRG1^+^CD8T cells in the tbLN (i.p., *n* = 7; all other groups: *n* = 6); Frequencies of G. KLRG1^+^CD4T cells (Control, *n* = 3; tbLN, *n *= 7; ntbLN, *n *= 6; i.p., *n* = 6) and H. KLRG1^+^CD8T cells in the spleen (Control, *n* = 6; tbLN, *n* = 7; ntbLN, *n* = 6; i.p., *n* = 7); Frequencies of I. CTLA4^+^CD8T (Control, *n* = 7; tbLN, *n* = 8; ntbLN,* n* = 6; i.p., *n* = 8) and J. PD1^+^ CD8T cells in the tbLN (Control, *n* = 7; tbLN, *n* = 7; ntbLN, *n* = 6; i.p., *n* = 8); Frequencies of K. CTLA4^+^CD8T (Control, *n *= 6; tbLN, *n* = 7; ntbLN, *n* = 7; i.p., *n* = 8) and L. PD1^+^CD8T cells in the spleen (Control, *n* = 6; tbLN, *n* = 8; ntbLN, *n* = 6; i.p., *n* = 8); M. IFNg (Control, *n* = 5; tbLN, *n* = 6; ntbLN, *n* = 5; i.p., *n* = 7) and N. IL10 levels in the tbLN (Control, *n = *5; tbLN, *n* = 6; ntbLN, *n* = 5; i.p., *n* = 7). Statistical analyses were done using ANOVA with Tukey’s test. *P < 0.05, **P < 0.01, and ****P < 0.0001. Data are represented as means ± SEM

To uncover the mechanistic underpinnings of tbLN specific blockade of CTLA4/CD80/86 axis resulting in a superior anti-tumor response, the immunome of mice treated with anti-CTLA4 through different drug delivery strategies was interrogated. CD4T cells were found to be significantly upregulated in the spleen, but not the tbLN of all anti-CTLA4 treated mice. However, upregulation was found to be the strongest in the case of tbLN treated mice. In addition, the tbLN and spleen were found to be remarkably enriched in CD8T cells in tbLN treated mice (Fig. 2 A – D). Particularly, greater fraction of KLRG1 (killer cell lectin-like receptor G1) expressing effector CD8T cell frequencies were found in both the spleen and the tbLN of tbLN treated mice (Fig. 2 E – H). CTLA4 was found to be significantly downregulated in both the tbLN and spleen of tbLN treated mice suggestive of superior drug distribution at site critical to generating anti-cancer effect upon localized delivery of anti-CTLA4 mAb (Fig. 2 I – L). In congruence with the expansion of CD4 and CD8T cells, IFNγ production in the LN was found to be significantly upregulated. Additionally, IL10 levels were found to be significantly upregulated in the tbLN group. Interestingly, IFNγ levels were found to correlate with IL10 levels (Fig. 2 M – N). In concert, these data suggest that reinvigoration of effector CD8T cells in the TME is key to eliciting a strong anti-tumor response.

**Fig. 3:**
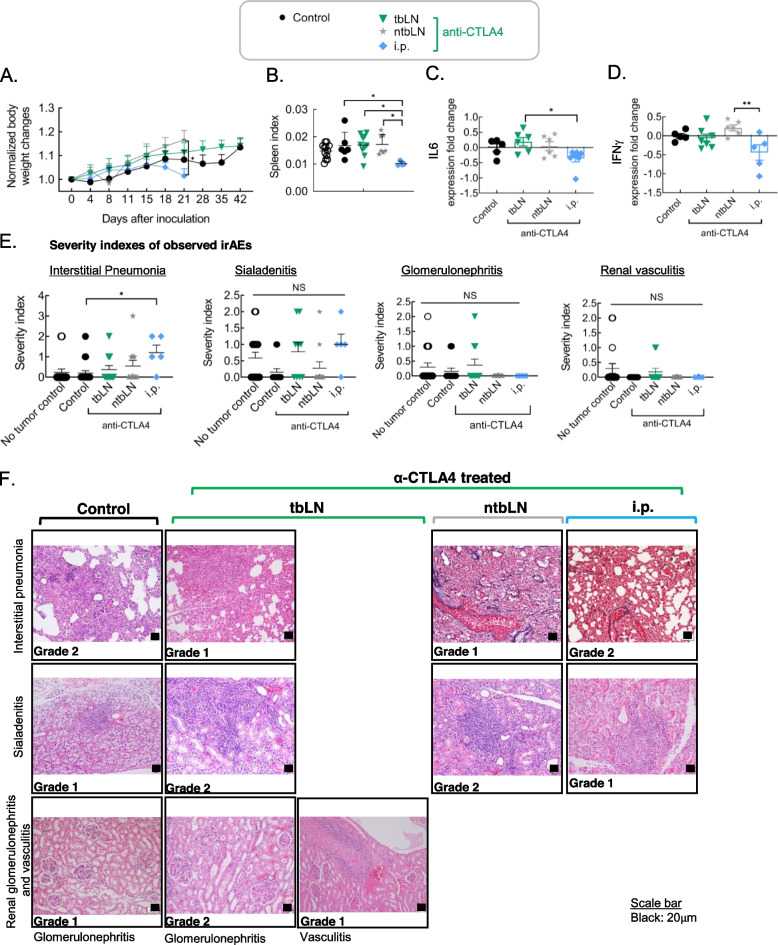
ICI-induced irAEs are amplified upon non-specific CTLA4 blockade. Adverse events were examined in unilaterally inoculated mice administered 5 mg/kg anti-CTLA4 into the tbLN or the ntbLN using a LDDS or through i.p. on day 4 and 8 post tumor inoculation. **A**. Body weight changes. Body weight was normalized to the day 0 value for each mouse. (Control, *n* = 14; tbLN, *n* = 10; ntbLN, *n* = 11; i.p., *n* = 8) **B**. Spleen index. Spleen index was obtained by dividing the weight of spleen by the body weight. (No tumor control, *n* = 12; Control, *n* = 6; tbLN, *n* = 10; ntbLN, *n* = 7; i.p., *n* = 4) **C**. Level of IL6 (Control, *n* = 5; tbLN, *n* = 6; ntbLN, *n* = 6; i.p., *n* = 7) and **D**. IFNγ in the spleen (Control, *n* = 5; tbLN,* n* = 7; ntbLN, *n* = 6; i.p., *n* = 5). **E**. Severity of different irAEs as confirmed by examination of stained tissue section. (Pneumonia: No tumor control, *n* = 17; Control, *n* = 20; tbLN, *n* = 11; ntbLN, *n* = 11; i.p., *n* = 5; Glomerulonephritis and Vasculitis: No tumor control, *n* = 17; Control, *n* = 13; tbLN, *n* = 11; ntbLN, *n* = 11; i.p., *n* = 5; Sialadenitis: No tumor control, *n* = 17; Control, *n* = 13; tbLN, *n = *9; ntbLN, *n *= 11; i.p., *n* = 5) **F**. HE (Hematoxylin and Eosin)- or EM (Elastica Masson)-stained tissue specimen showing frequently observed irAEs in the case of anti-CTLA4 administered using different strategies. Statistical analyses were done using ANOVA with Tukey’s test. *P < 0.05, **P < 0.01, and ****P < 0.0001. Data are represented as means ± SEM

Next, we sought to determine whether ICI administration route had any impact on ICI-induced toxicity. Day 15 onwards, body weight in the i.p. group was found to be declining (Fig. 3A). Additionally, the spleen index of the i.p. group was also found to be markedly low, indicating a decrease in spleen cellularity (Fig. 3B). Consequently, significant down regulation of IFNγ and IL6 was observed in this group (Fig. 3C – D). Furthermore, ICI administration led to the appearance of a variety of irAEs, recapitulating clinical scenarios, where onset of severe irAEs often results in stalling or in extreme cases halting of immunotherapy.

CTLA4 targeted therapy was found to result in the development of interstitial pneumonia, glomerulonephritis, renal vasculitis, sialadenitis and rarely cases of hepatitis, colitis, gastritis, and arthritis with varying severity in each group (Fig. 3 E – F). Particularly, non-specific CTLA4 inhibition (i.p.) was found to result in interstitial pneumonia of higher severity despite shorter observation period, implying that CTLA4 inhibition systemically or in non-tumor environments is detrimental as it yields more off target effects (Fig. 3 E – F). Concordantly, macrophage infiltration was observed in pneumonia positive tissues.

Taken together, these data demonstrate tumor-microenvironment-directed ICB can potentiate therapeutic response while minimizing irAEs. In contrast non-specific administration of ICI can not only drastically reduce quality of life but in fact cut it short as well (Fig. 1 I). In the case of delivery of anti-CTLA4 to tbLN, it is likely that the tumor tissue outcompetes the non-diseased tissue in terms of ICI availability, thereby minimizing unwarranted off-target effects. Therefore, ICI-administration site is a critical variable dictating both the therapeutic response and ICI-associated toxicity.

### anti-CTLA4 mAb administered using LDDS to tumor bearing LN can inhibit distant metastasis

**Fig. 4:**
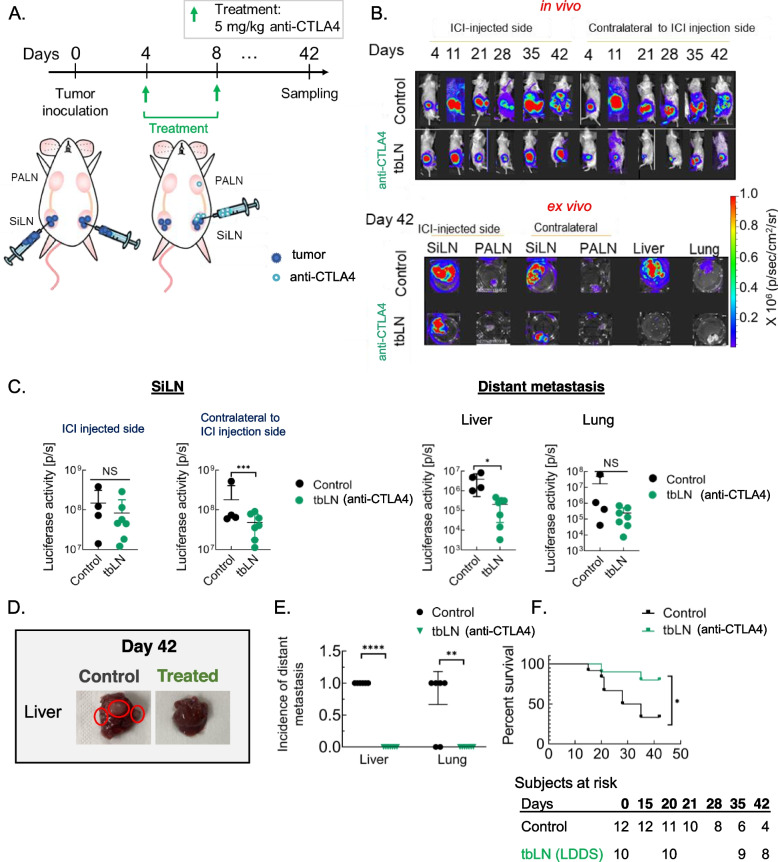
Intra-nodal administration of low-dose anti-CTLA4 mAb can prevent distant metastasis. Bilaterally inoculated MXH10/Mo/lpr were administered 5 mg/kg anti-CTLA4 to tbLN on day 4 and 8 to one of the two tumor-bearing SiLN. **A**. Experimental outline **B**. Two sets of representative bioluminescent images: in vivo (upper panel) and ex vivo (lower panel). **C**. ex vivo luciferase activity of the SiLN, Liver and Lung. (Control, *n* = 4; tbLN, *n* = 7) **D**. Representative image of control and treated liver. Red circle: metastatic nodules. **E**. Incidence of liver and lung metastases (Control, *n* = 6; tbLN, *n* = 7) **F**. Survival curves of mice. (Control, *n = *12; tbLN, *n* = 10). Statistical analyses were done using ANOVA with Tukey’s test or unpaired two-tailed student’s t-test. Log-rank (Mantel-Cox) test for survival curves. *P < 0.05, **P < 0.01, and ****P < 0.0001. Data are represented as means ± SEM

CTLA4 blockade through administration of anti-CTLA4 mAb via LDDS to tumor bearing LN was found to successfully lead to total tumor rejection in about 67% of treated subjects (Fig. 1H). To ascertain whether CTLA4 blockade could prevent and/or treat distant metastasis, cumulative 10 mg/kg dose of α-CTLA4 mAb was administered to one of the two tumor-bearing LNs of bilaterally inoculated mice (Fig. 4A). Of note, incidence of distant metastasis as well as the metastatic burden, is greater in bilaterally inoculated mice as compared to unilaterally inoculated mouse model (table S4, Fig. 1 G). Contrary to our expectations, mild tumor regression was observed in the SiLN contralateral to the anti-CTLA4 injected SiLN but not the anti-CTLA4 injected SiLN (Fig. 4 B – C, Fig. S3 A). Additionally, treatment resulted in total prevention of lung and liver metastasis (67% incidence of lung and 100% incidence of liver metastases in control group vs 0% in treated group) as evidenced by lower ex vivo luciferase activity and incidence of metastases in these organs (Fig. 4 C – E). It is, however, important to note that in the case of ex vivo bioluminescent imaging, sample size of control group was low for conclusive statistical interpretation due to a high attrition rate, but the conclusion made was backed by histological and survival analyses. Overall survival was found to be substantially higher in the treated set (80% in treated vs 27% in untreated control) (Fig. 4 F). However, as is the case with ICI therapy, irAEs were observed in anti-CTLA4 treated mice (Fig. S3 A – B). Importantly, greater severity of interstitial pneumonia was noted in the control group as opposed to the no tumor control group, likely due to high tumor burden raising the possibility of paraneoplastic syndrome [52].

### Therapeutic efficacy of CTLA4 mAb is directly proportional to the dosage administered

**Fig. 5:**
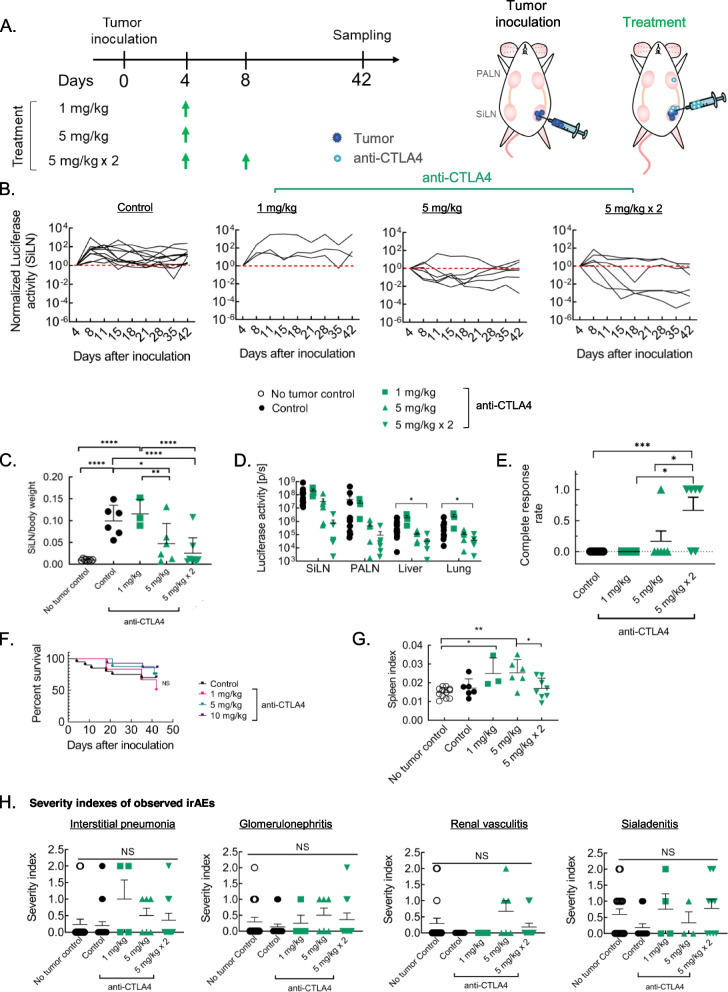
tbLN-directed CTLA4 blockade demonstrates dose-dependency in tumor inhibition but not in severity of irAEs. To examine the dose-dependency of CTLA4 mAb on therapeutic efficacy and toxicity, 1 mg/kg or 5 mg/kg on day 4 or cumulative dosage 10 mg/kg (5 mg/kg × 2) day 4 and 8 was administered to tbLN of unilaterally inoculated mice. **A**. Experiment outline **B**. Normalized SiLN in vivo luciferase activity of individual mice. (Control, *n* = 12; 1 mg/kg,* n* = 3; 5 mg/kg, *n* = 6; 10 mg/kg, *n* = 6) **C**. tumor inoculated SiLN weight normalized to mouse body weight. (Control, *n* = 12; 1 mg/kg, *n* = 3; 5 mg/kg, *n* = 6; 10 mg/kg, *n* = 6) **D**. ex vivo luciferase activity of the SiLN, PALN, Liver and lung as measured on day 42 post tumor inoculation. (Control, *n* = 12; 1 mg/kg, *n* = 3; 5 mg/kg, *n* = 6; 10 mg/kg, *n* = 6) **E**. Complete response rate (Control, *n* = 12; 1 mg/kg, *n* = 3; 5 mg/kg, *n* = 6; 10 mg/kg, *n* = 6) **F**. Plot showing overall survival of mice in each group. (Control, *n* = 20; 1 mg/kg, *n* = 6; 5 mg/kg, *n* = 8; 10 mg/kg, *n* = 14) **G**. Spleen index of each group. Spleen index was calculated by dividing the spleen weight at experimental endpoint by the mouse body weight. (No tumor control, *n* = 12; Control, *n* = 6; 1 mg/kg, *n* = 3; 5 mg/kg, *n* = 6; 10 mg/kg, *n* = 8) **H**. Severity of different irAEs (Pneumonia: No tumor control, *n* = 17; Control, *n* = 20; 1 mg/kg,* n* = 4; 5 mg/kg, *n* = 6; 10 mg/kg, *n* = 11; Glomerulonephritis and Vasculitis: No tumor control, *n* = 17; Control, *n* = 13; 1 mg/kg, *n* = 4; 5 mg/kg, *n* = 6; 10 mg/kg, *n* = 11; Sialadenitis: No tumor control, *n* = 17; Control, *n* = 13; 1 mg/kg,* n* = 4; 5 mg/kg, *n* = 2; 10 mg/kg, *n* = 9). Statistical analyses were done using ANOVA with Tukey’s test. *P < 0.05, **P < 0.01, and ****P < 0.0001. Data are represented as means ± SEM

Next, the sensitivity of therapeutic efficacy and irAE to concentration of anti-CTLA4 mAb administered was explored (Fig. 5 A). In line with previously published reports [53], a clear dose-dependent relationship was observed between therapeutic efficacy and anti-CTLA4 dosage, with administration of a cumulative 10 mg/kg resulting in remarkable tumor inhibition (Fig. 5 B – E, Fig. S4). Consequently, overall survival at this concentration was better than the other groups (Fig. 5 F). Therapeutic efficacy of anti-CTLA4 mAb was found to drop significantly when dosage administered was sub-optimal. At optimal concentration, the spleen index was found to be similar to no tumor control mice but significantly lower than that of mice treated with lower doses of anti-CTLA4 (Fig. 5 G). In the context of irAEs, no such trend was apparent (Fig. 5 H, Fig. S4). In agreement with previously published reports, no correlation was found between therapeutic efficacy and anti-CTLA4 blockade induced irAEs [54].

### α-PD1 mAb has limited potential to treat tumor bearing SiLN

To investigate the impact of PD1/PDL1 axis blockade in vivo, anti-PD1 mAb was administered locally to tbLN at different concentrations (Fig. S5 A). 1 mg/kg was found to be the optimal concentration. Mean ex vivo luciferase activity of the SiLN, PALN, lung and liver were found to be the least at this concentration. Overall survival and complete response rate at this concentration were found to be the highest. Above and below this optimal concentration, no additional therapeutic benefit was observed (Fig. S5 B – F). Histological findings were in concordance with the imaging data (Fig. S6). Spleen index was found to be the least at this optimal concentration in comparison to other treated groups. However, it was still similar to that of untreated tumor-free MXH10/Mo/lpr mice (Fig. S5 G). Development of glomerulonephritis was found to be particularly sensitive to anti-PD1 dosage (Fig. S5 H, Fig. S6).

**Fig. 6:**
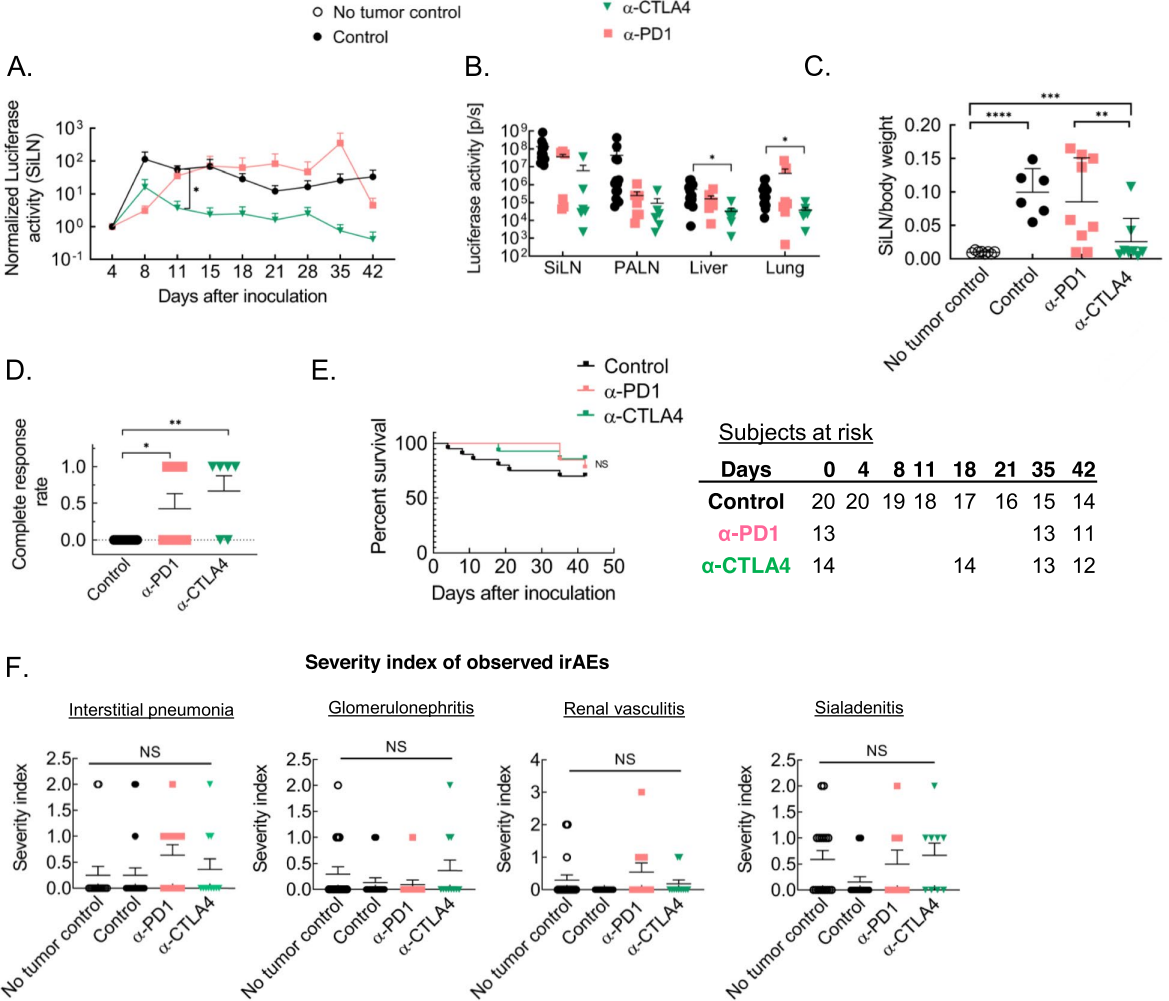
Comparison of tumor inhibition and irAE severity caused by tbLN-directed PD1 and CTLA4 blockade in a unilaterally inoculated mouse model. **A**. Normalized SiLN luciferase activity (Control, *n* = 12; anti-PD1, *n* = 6; anti-CTLA4, *n* = 6) **B**. ex vivo luciferase activity of the SiLN, PALN, liver and lung as measured on day 42 post tumor inoculation. (Control, *n* = 12; anti-PD1, *n* = 7; anti-CTLA4, *n* = 6) **C**. SiLN/body weight at pre-determined experimental endpoint (No tumor control, *n* = 12; Control, *n* = 6; anti-PD1, *n* = 9; anti-CTLA4, *n* = 8) **D.** Complete response rates (Control, *n* = 12; anti-PD1, *n *= 7; anti-CTLA4, *n *= 6) E. Survival plots (Control, *n* = 20; anti-PD1, *n* = 13; anti-CTLA4, *n* = 14) **E**. Severity index of different irAEs (Interstitial pneumonia: No tumor control, *n* = 17; Control, *n* = 20; anti-PD1, *n* = 11; anti-CTLA4, *n* = 11; Glomerulonephritis and Vasculitis: No tumor control, *n* = 17; Control,* n* = 13; anti-PD1, *n* = 11; anti-CTLA4, *n* = 11; Sialadenitis: No tumor control, *n* = 17; Control, *n* = 13; anti-PD1, *n* = 8; anti-CTLA4, *n* = 9). Statistical analyses were done using ANOVA with Tukey’s test. Log-rank (Mantel-Cox) test for survival curves. *P < 0.05, **P < 0.01, and ****P < 0.0001. Data are represented as means ± SEM

However, in comparison to optimally dosed anti-CTLA4 mAb administered via LDDS to tbLN, PD1 blockade was found to result in marginally weaker tumor inhibition as is evidenced by its relatively higher ex vivo luciferase activity, normalized in vivo luciferase activity and lower complete response rate and overall survival (Fig. 6 A – D). Although not statistically significant, overall survival was also greater in anti-CTLA4 treated mice (Fig. 1, Fig. S5, Fig. 6 E). However, a heterogeneity in responses and prevalence of irAEs was found to be common to both tbLN-directed PD1 and CTLA4 blockade therapies (Fig. 6F). Severity of interstitial pneumonia induced by tbLN targeted PD1 blockade was found to be greater than in the case of tbLN targeted CTLA4 blockade.

## Discussion

ICIs have demonstrated encouraging response rates, resulting in their FDA approval as a first line treatment in a variety of malignancies [55–57]. However, a vast majority of patients show primary resistance to ICIs [15–17, 58]. Another roadblock to their widespread usage is that their responses are often mired by unwarranted off-target effects, often resulting in stalling or cessation of therapy [13, 53, 56, 58, 59]. Additionally, there is evidence to suggest that steroids administered for the management of ICI-induced irAEs may interfere with the therapeutic response by reducing T cell infiltration [60]. Therefore, in the present study we investigated the consequences of TME-specific (intra-nodal to tbLN) and non-specific blockade (intra-nodal to ntbLN, and i.p.) in a bid to identify optimal ICI delivery strategy capable of potentiating therapeutic response while attenuating off-target events.

To the best of our knowledge, this is the first published report investigating the therapeutic response and immune related side effects induced by ICI in a mouse model of lymph node metastasis capable of recapitulating clinically observed ICI-induced irAEs, particularly, ICI-induced interstitial pneumonia.

Herein, we demonstrated the superior tumor inhibition potency of drug injected to the tbLN for the treatment of lymph node metastases and the prevention and/or treatment of systemic metastases. This is in line with previous published reports that demonstrated that systemic or non-specific administration of ICIs elicits inferior therapeutic response in case of other types of malignancies [61–63]. Significant upregulation of CD8T cells, and particularly, effector memory CD8T cells (KLRG1^+^CD8T cells), capable of killing tumor cells in a granzyme B dependent manner [64], in the tbLN and spleen with significant upregulation of IFNγ and IL10 in the tbLN was noted mice in mice administered anti-CTLA4 to the tbLN. This implicates that both increased frequency of tumor infiltrating CD8 lymphocytes, and the reinvigoration and sustained activation of CD8T cells were critical to the generation of a robust and sustained anti-tumor response [65]. It is important to note that although IL10 is widely regarded as an immune suppressive cytokine, it is a highly pleiotropic cytokine whose activity is context dependent [66, 67]. IL10 has been shown to upregulate the production of cytotoxic enzymes and IFNγ in tumor infiltrating CD8 + T lymphocytes, thereby inducing antigen-presentation. Its upregulation might thus be critical to overcoming immunological barriers to the activation of effector T cell functions in the present context [68, 69]. Importantly, IL10 was found to be significantly downregulated in the tbLN in mice that were administered anti-CTLA4 i.p. indicating the absence of IL10 mediated CD8T cell activation. Significant upregulation of CD8T cells and its effector function, and downregulation of its CTLA4 expression, in the case of tbLN specific administration of anti-CTLA4 may be reflective of the superior pharmacokinetics of intranodal drug administration as opposed to systemic approaches [9, 60]. In such a scenario, tumor can outcompete non-diseased tissues in terms of drug availability. Importantly, tbLN-directed CTLA4 blockade was also confirmed to result in the prevention/treatment of systemic metastases, significantly prolonging survival. Additionally, while no therapeutic response was observed in the anti-CTLA4 mAb administered SiLN, a mild treatment effect was observed in the contralateral SiLN, raising the possibility of a functional activation of CD8T cells in these tissues. This suggests the possibility of a systemic activation of a functional anti-tumor response upon tbLN-directed CTLA4 blockade, hinting at a possibility of optimal ICB regimen (multi-drug and/or multi-site or multi-dosed) being potent enough to induce a complete remission. This is in line with previous reports in a mouse model of implanted B16F10 cells treated intratumorally with anti-PD1 and anti-CTLA4 mAb [70]. However, further studies need to be undertaken to understand the surprising lack of tumor control in ICI injection site, in light of its apparent efficacy in the contralateral side. Additionally, studies to elucidate the mechanisms inducing this abscopal-like phenomenon in our present model are needed.

Clinically, around 64.2% of patients treated with CTLA4 antagonists alone develop irAE [33, 34]. Recent reports cite that ICI-induced irAEs may be caused by a variety of factors like boosting of pre-existing subclinical autoimmunity or vulnerable genetic background [41, 48, 49, 71]. Importantly, in clinical settings, certain irAEs are underreported and/or understudied due to under-diagnoses of asymptomatic and difficult to diagnose irAEs like pneumonitis, [38] and/or underreporting due to lack of diagnostic technique in clinical trial inclusion criteria, like in the case of renal events [72]. In case of MXH10/Mo/lpr mice treated with CTLA4 antagonist, irAEs of varying severity, particularly, interstitial pneumonia was observed. Importantly, we reported that non-specific administration results in the exacerbation of irAEs, likely due to non-specific immune activation resulting in autoimmunity [73, 74]. i.p. administration was found to result in severe spleen atrophy as a consequence of which IFNγ production and IL6 production were found to be remarkably low. Such a correlation between decreased IFNγ, IL6 levels and spleen atrophy has previously been reported in an experimental stroke model, where increased mortality caused by pneumonia was noted [75].

One limitation of the present study is that i.p., and not i.v. was used as a proxy for systemic delivery to study the impact of systemic CTLA4 blockade. While i.p. delivery is widely used in preclinical studies to approximate systemic drug delivery methodologies [63, 70, 76], it is i.v. that is commonly used in clinical settings. Previous study in murine model of cancer reported comparable, if not lower, retention of IgG1 in the spleen, liver and muscle of i.p. administered mice in comparison to i.v. administered mice, over a period 120 h [77]. Based on this finding, we expect i.v. delivery of anti-CTLA4 mAb to further exacerbate spleen pathology. Another study reported similar levels of Docetaxel in spleen of i.p. and i.v. delivered mice. However, there were subtle differences in their retention in peritoneal tumors, reflective of differences in metabolism and pharmacokinetics of i.p. and i.v. administered docetaxel [78]. Therefore, further preclinical studies focusing on the pharmacokinetics and pharmacodynamics of anti-CTLA4 mAbs are mandated for the translation of the findings of the present study.

Of clinically observed ICI-induced irAEs, perhaps the most important observation in ICI-treated MXH10/Mo/lpr was interstitial pneumonia. Particularly, high severity of interstitial pneumonia was induced by ICI treatment in MXH10/Mo/lpr mice in a setting of established tumor to lymph node. Even though the incidence of clinically observed ICI-induced pneumonia is low, it is one of the few irAEs that has been associated with drug-related death [38–42, 79]. Importantly, the risk factors of ICI-induced interstitial pneumonitis are not yet clearly defined [38, 39, 41, 79, 80]. Severe pneumonia was observed upon non-specific administration of anti-CTLA4 i.p. or to ntbLN, likely due to disruption of normal immune system homeostasis by ICI at a site critical to mounting immune response because of non-specific activation [60]. However further studies in the background of MXH10/Mo/lpr mice are warranted for the identification of genetic drivers, and cellular and molecular mediators of interstitial pneumonia. Particularly, identification of biomarkers in patients can be guided by preliminary data based on additional preclinical studies utilizing this model. Such studies may aid in the identification of patients with a predisposition to developing ICI-induced interstitial pneumonia and thus inform the design of patient centered N-of-one personalized clinical studies [81]. Such studies are instrumental for the development of personalized immunotherapy [47].tbLN-directed PD1 blockade was not found to be as efficacious as tbLN-directed CTLA4 blockade. The higher therapeutic sensitivity to tbLN-directed anti-CTLA4 as opposed to tbLN-directed anti-PD1 can be explained by its spatial regulation. Prolonged retention of anti-CTLA4 in the tbLN microenvironment, where the T cell priming is known to occur, is therefore crucial to its therapeutic response [22]. However, this leaves a window open for the exploration of combination approaches of tbLN-directed CTLA4 blockade with optimally targeted PD1 blockade, banking on synergistic or additive effects of the two ICIs.

Indeed, clinical findings have validated the superior therapeutic efficacy of combining ICIs in the context of melanoma, renal cell carcinoma, and metastatic colorectal cancer [58, 82]. Importantly, however, the dysregulation of a myriad of processes, and not just checkpoints, is responsible for growth and sustenance of tumors. A complex crosstalk facilitates processes such as angiogenesis, lymphangiogenesis, immune evasion and suppression, etc. that allow cancer cells to thrive. Consequently, a recent study reported remarkable success upon simultaneous application of ICB with CAR (Chimeric antigen receptor) T cell therapy by virtue of ICI’s ability to reverse the immunosuppressive microenvironment generated by CAR T cell therapy [82, 83]. Another study demonstrated the capacity of capecitabine, a chemotherapeutic agent, to downregulate CTLA4 expression on CRC (colorectal cancer) tissues, shedding light on its immunomodulatory capacity in addition to its previously known disruption of DNA synthesis [84]. Carter et. al. reported improved efficacy upon combination of Ipilimumab (anti-CTLA-4 mAb) and Bevacizumab (anti-VEGF mAb) in glioblastoma patients [85]. These findings support further studies utilizing novel combinations to improve the sensitivity of cancer cells to ICIs. However, key to achieving widespread success with novel immunomodulating combinations is a thorough assessment of biomarkers from primary TME, tumor draining lymph nodes and peripheral sites that can inform the best combination of systemic, intratumoral or intranodal approaches in order to maximize tumor control while simultaneously minimizing synergistic toxicity [22]. Herein, using a CTLA4 blocker, we have demonstrated superior tumor inhibition with minimal irAE. The findings of the present study can inform optimal combinatorial strategies involving FDA-approved anti-CTLA4 mAb, ipilimumab and tremelimumab.

One limitation of the present study is that active drug delivery approaches are mandated for the expansion of proposed drug-delivery methodology to inaccessible MLNs.

However, while the present preclinical study does provide critical insights, it is important to note that murine models of cancer do not fully recapitulate the complexity and heterogeneity of human tumors and/or immune biology [86]. New techniques/technologies that better reflect the biology of human cancer immunology are needed. Careful consideration in clinical trial design and inclusion criteria, based on extensive genomic, transcriptomic, immunomic, proteomic, and metabolomic profiling is mandated to effectively translate these findings from bench to bedside [81].

But, in view of likelihood of fatal consequences of lymphadenectomy [6], inefficiency of systemic drug delivery approaches [9, 21, 87], low economic burden, FDA approval [11], and simplicity of translation of present approach, we believe the findings of the present study have great clinical utility in the context of patients of N positive status with accessible LNs.

## Conclusion

In summary, tbLN-specific CTLA4 blockade effectively limits LN metastasis and prevents systemic metastasis, thereby prolonging survival. Non-specific CTLA4 blockade was found to be associated with high morbidity and mortality due to a high severity of ICB-induced irAEs.

## Supplementary Information

**Additional file 1: Supplementary table S1.** Strain distribution patterns of MXH10/Mo-*lpr/lpr *mouse by microsatellite markers. **Supplementary table S2.** irAE grading criteria.** Supplementary Table S3.** Primers and probes for RT-PCR. **Supplementary table S4.** Incidence of distant metastasis in murine models of lymph node metastasis. **Figure S1.** Interstitial pneumonia grading. **Figure S2.** Histological evaluation of therapeutic efficacy and irAEs induced by tbLN-directed CTLA4 blockade. **Figure S3.** ICI induced therapy and toxicity in bilaterally inoculated mice. **Figure S4.** Histological evaluation of low doses of anti-CTLA4 mAb administered to the tbLN in a unilaterally inoculated mouse model. **Figure S5.** Anti-PD1 delivery through LDDS is sensitive to administered dosage. **Figure S6.** Histological evaluation of mice treated with different dosage of anti-PD1 using LDDS.

## Data Availability

The datasets used and/or analyzed during the current study are available from the corresponding author on reasonable request.

## Abbreviations

CAR: Chimeric antigen receptor
CRC: Colorectal cancer
DC: Dendritic cell
EM: Elastica masson
HE: Hematoxylin and eosin
ICB: Immune checkpoint blockade
i*.*p*.*: Intraperitoneal
irAE: Immune-related adverse event
IVIS: in vivo Imaging system
LN: Lymph node
LDDS: Lymphatic drug delivery system
MLN: Metastatic lymph node
MRL/lpr: MRL/Mp-*lpr/lpr*
MXH10/Mo/lpr: MXH10/Mo-*lpr/lpr*
ntbLN: Non-tumor bearing lymph node
PALN: Proper axillary lymph node
SiLN: Subiliac lymph node
tbLN: Tumor-bearing lymph node
